# CDKN2B Inhibits Vascular Smooth Muscle Phenotypic Switching in Corpus Spongiosum Surrounding the Urethral Plate in Hypospadias

**DOI:** 10.3390/biomedicines14010032

**Published:** 2025-12-23

**Authors:** Jiayao Huang, Zihan Xu, Jiacheng Huang, Xiaoqin Yin, Yichen Huang, Fang Chen

**Affiliations:** Department of Urology, Shanghai Children’s Hospital, School of Medicine, Shanghai Jiao Tong University, Shanghai 200062, China; dr_huangjiayao@163.com (J.H.);

**Keywords:** hypospadias, corpus spongiosum, phenotype switching, CDKN2B

## Abstract

**Objective:** Phenotypic switching of vascular smooth muscle cells (VSMCs) in the corpus spongiosum may contribute to abnormal urethral development in hypospadias, but the underlying molecular regulators remain unclear. This study aimed to identify hub genes associated with VSMCs phenotypic switching in the corpus spongiosum using RNA sequencing and Weighted Gene Co-expression Network Analysis (WGCNA), and to functionally characterize the top candidate gene *CDKN2B*. **Methods**: Corpus spongiosum tissue samples were collected from seven patients with proximal hypospadias and five patients with urethral stricture (control group). The expression of the VSMCs contractile markers Calponin 1 and α-SMA, and the secretory marker OPN, was evaluated by qRT-PCR and Western blotting to assess VSMCs phenotypic state. RNA sequencing and Weighted Gene Co-expression Network Analysis (WGCNA) were performed to identify hub genes, which were then validated by qRT-PCR. Primary VSMCs were isolated from corpus spongiosum tissue and transduced with lentiviral vectors to either suppress or overexpress *CDKN2B*. Changes in VSMC marker expression and in key signaling pathways associated with phenotypic switching—specifically *TGF/Smad* and *SRF/MYOCD*—were analyzed using qRT-PCR and Western blotting. **Results**: In hypospadias tissue, the decreased expression of α-SMA and Calponin 1, together with increased OPN, indicated a shift in VSMCs from a contractile to a secretory phenotype. RNA-seq and WGCNA identified 11 differentially expressed genes, among which *CDKN2B* showed a marked downregulation in hypospadias samples. In control VSMCs, *CDKN2B* inhibition led to reduced α-SMA and Calponin 1, elevated OPN, and suppressed activity of *TGF/Smad* and *SRF/MYOCD* signaling. Conversely, *CDKN2B* overexpression in VSMCs from hypospadias samples restored α-SMA and Calponin 1 expression, decreased OPN, and enhanced *TGF/Smad* and *SRF/MYOCD* pathway activation. **Conclusions**: VSMCs in the corpus spongiosum surrounding the urethral plate in hypospadias undergo a transition from a contractile to a secretory phenotype. *CDKN2B* emerges from unbiased transcriptomic screening as a key hub gene and functions as a critical regulator of this process, maintaining the contractile phenotype by modulating canonical *TGF/Smad* and *SRF/MYOCD* signaling. The *CDKN2B–TGF/Smad* axis may represent a central pathway linking VSMC phenotypic switching to abnormal vascular remodeling in hypospadias.

## 1. Introduction

Hypospadias is a common congenital malformation of the male genitourinary system, affecting approximately 1 in 300 live births. It is characterized by an abnormally positioned urethral opening and underdevelopment of the ventral aspect of the penis [1], often accompanied by structural abnormalities of the corpus spongiosum surrounding the urethral plate.

Gearhart et al. [2] described morphological defects in the corpus spongiosum, noting that its distal portion forms a bifurcated columnar structure on both sides of the urethral plate, extending distally to merge with the glans. Subsequent studies by Baskin [3] and Putte [4] revealed irregular dilation of vascular sinuses in the fetal corpus spongiosum and glans. Our previous work further demonstrated that a key pathological feature of the urethral corpus spongiosum in hypospadias is thickening of the vascular smooth muscle layer [5], which was similar to the pathological changes seen in erectile dysfunction (ED), where vascular density decreases and vessel walls become thickened. These findings suggest that abnormalities in vascular smooth muscle cells (VSMCs) may contribute to the pathogenesis of hypospadias

VSMCs are highly plastic cells capable of reversible transitions between contractile and synthetic phenotypes in response to environmental cues. In our earlier research, we observed phenotypic alterations of VSMCs within the urethral corpus spongiosum in hypospadias, supporting the notion that such phenotypic modulation may underlie the abnormal structure of the corpus spongiosum [6]. Our previous study mainly described phenotypic alterations of vascular smooth muscle cells in hypospadias [6]. In the present work, we extend these observations by using RNA sequencing combined with weighted gene co-expression network analysis (WGCNA) to explore the upstream molecular mechanisms underlying this phenotypic switch.

Based on these findings, we hypothesized that VSMC phenotypic switching plays a critical role in the abnormal remodeling of the corpus spongiosum in hypospadias. To unbiasedly explore upstream regulators of this process, we performed RNA sequencing (RNA-seq) to compare gene expression profiles between hypospadias and control tissues, and applied Weighted Gene Co-expression Network Analysis (WGCNA) to identify gene modules and hub genes associated with the hypospadias phenotype. We then focused on the cyclin-dependent kinase inhibitor *CDKN2B*, a hub gene within the most strongly hypospadias-associated module, and investigated its role in regulating VSMCs phenotypic transformation and associated signaling pathways.

## 2. Materials and Methods

### 2.1. Sample Collection

This study was approved by the Ethics Committee of Shanghai Children’s Hospital (No. 2021RY033-E01). The experimental group comprised seven patients with scrotal hypospadias (mean age: 29.14 ± 15.27 months). Corpus spongiosum tissue was obtained from both sides of the forked urethral plate during surgical repair. The control group included five patients with urethral stricture secondary to trauma (mean age: 15.80 ± 8.81 years); samples of normal corpus spongiosum tissue adjacent to the stricture site were collected as control group [5].

### 2.2. RNA Sequencing (RNA-seq) and Weighted Gene Co-Expression Network Analysis (WGCNA)

Total RNA was extracted from corpus spongiosum tissue using TRIzol reagent (Invitrogen, CA, USA). RNA libraries were constructed using the TruSeq RNA Library Prep Kit v2 (Illumina, Inc, San Diego, CA, USA) following the manufacturer’s protocol and sequenced on an Illumina NovaSeq 6000 platform with 150 bp paired-end reads.

Raw sequencing reads were processed using Skewer to remove adaptor sequences and low-quality fragments. Read quality was assessed with FastQC (www.bioinformatics.babraham.ac.uk/projects/fastqc/, accessed on 1 January 2023). Clean reads were aligned to the human reference genome (GRCh38) using STAR, and transcript assembly was performed with StringTie (v2.2.1) using Ensembl (v110) annotations. Gene expression levels were quantified as FPKM (Fragments Per Kilobase of transcript per Million mapped fragments).

Differential gene expression was analyzed using the DESeq2 package in R (v4.3.2.) Genes with |log_2_ fold change| ≥ 1 and adjusted *p* < 0.05 (false discovery rate, FDR) were considered significantly differentially expressed. Gene Ontology (GO) and KEGG pathway analyses were performed to explore functional enrichment of the identified genes.

The present study employed the WGCNA package (v1.73) in R software to cluster functional modules in RNA-seq data, utilizing a defined cut-off height of 0.25 to merge similar modules. The co-expression network was integrated with group phenotypes (control vs. hypospadias), and hub genes with high modularity were identified through modular-trait correlation analysis. A heatmap was utilized to visualize the correlation between gene modules and clinical traits, followed by the identification of modules associated with co-expression patterns (turquoise module) and phenotypes. The shared genes between DEGs and WGCNA hub genes were obtained through the utilization of Venny.2, version 2.1.0, an online tool for VENN analysis. The identification of crucial pathways involved in phenotypic switching was achieved by analyzing the shared genes, utilizing Metascape (http://metascape.org/, accessed on 1 January 2023).

### 2.3. Histological Studies and Immunohistochemical Staining

The tissue samples were fixed in a 4% paraformaldehyde solution for 6 h, then embedded in paraffin and consecutively sectioned. To observe the structure of corpus spongiosum, HE staining was performed. The expression and distribution of the target protein were observed through immunohistochemical staining using the DAB method.

### 2.4. Real-Time Fluorescent Quantitative PCR (qRT-PCR)

Total RNA was extracted using TRIzol reagent (Invitrogen) and reverse-transcribed to cDNA using a reverse transcription kit (Takara, kusatsu shiga, Japan). qRT-PCR was performed using SYBR Green Master Mix in a 10 μL reaction system under the following cycling conditions: 95 °C for 10 min, followed by 40 cycles of 95 °C for 10 s, 60 °C for 30 s, and 72 °C for 30 s.

Primer sequences for target genes (α-SMA, Calponin 1, OPN, SRF, MYOCD, SMAD2, SMAD3, TGF-β1, FOXO4, and KLF4) were designed based on published human sequences (Table S1).

### 2.5. Western Blot

Tissue samples were lysed in RIPA buffer and centrifuged to obtain protein extracts. Protein concentrations were determined using the BCA assay. Equal amounts of protein were separated by SDS–PAGE, transferred to PVDF membranes, and blocked for 1 h at room temperature. Membranes were incubated overnight at 4 °C with primary antibodies against CDKN2B, β-actin, GAPDH, α-SMA, Calponin 1, OPN, TGF-β1, Smad2, and Smad3 (Abcam or CST; dilutions 1:500–1:1000). After incubation with HRP-conjugated secondary antibodies, signals were detected using an ECL chemiluminescence system, and relative expression levels were quantified by grayscale analysis

### 2.6. Primary Cell Culture

Fresh corpus spongiosum tissue was rinsed in PBS (Gibco, NY, USA), minced into approximately 1 mm^3^ fragments, and digested with type IV collagenase for 30 min at 37 °C. The digested mixture was centrifuged, washed with prewarmed medium, and transferred to 24-well plates containing a commercially available smooth muscle cell medium (Smooth Muscle Cell Medium, ScienCell, Carlsbad, CA, USA) supplemented with the manufacturer’s smooth muscle growth supplement and 1% penicillin–streptomycin. Cultures were maintained at 37 °C in a humidified atmosphere of 5% CO_2_. The medium was changed every two days, and cells were subcultured upon reaching 80–90% confluence.

### 2.7. Cell Immunofluorescence Staining

The protocol involved fixing cells with 4% paraformaldehyde, permeabilizing them with 0.5% Triton X-100, incubating with a primary antibody against α-SMA (a marker of smooth muscle cells), and then incubating with a secondary antibody conjugated with a fluorescent dye. The nuclei were stained with DAPI, a fluorescent DNA-binding dye. The cells were then visualized under a fluorescence microscope, which allows for the visualization of the fluorescent signals emitted by the secondary antibody and the DAPI stain.

### 2.8. Cell Transfection

Lentiviral vectors encoding *CDKN2B* overexpression (oeRNA) or *CDKN2B* knockdown (shRNA) constructs were obtained from Shanghai Hanheng Biotechnology Co., Ltd. Lentiviral particles were produced by co-transfecting the constructs with packaging and envelope plasmids into 293T cells. Primary VSMCs were infected with lentivirus at a multiplicity of infection (MOI) of 1 (1 × 10^8^ TU/mL). The experimental groups were as follows:

Control group (blank lentiviral control);
ko-CDKN2B group (*CDKN2B* shRNA-infected controls);
Hypospadias group (blank lentiviral infection in hypospadias-derived VSMCs);
oe-CDKN2B group (*CDKN2B* overexpression in hypospadias-derived VSMCs).

Thus, *CDKN2B* was knocked down in control-derived VSMCs and overexpressed in hypospadias-derived VSMCs to examine loss- and gain-of-function effects in complementary cellular backgrounds.

### 2.9. TGF-β1 Supplement

After establishing primary cultures of hypospadias-derived VSMCs, recombinant TGF-β1 protein (Abclonal, Wuhan, China) was added to the culture medium at a final concentration of 1 μg/mL. Cells were maintained for 7 days to evaluate the effect of exogenous TGF-β1 stimulation [7].

### 2.10. Statistical Analysis

All data are expressed as the mean ± standard deviation (SD). For normally distributed data with homogenous variance, comparisons between two groups were performed using an unpaired Student’s *t*-test, and multiple-group comparisons were analyzed by one-way ANOVA. Nonparametric data were analyzed using the Mann–Whitney U test. A two-sided *p* < 0.05 was considered statistically significant. Statistical analyses were performed using SPSS 23.0 (IBM Corp., Armonk, NY, USA).

## 3. Results

### 3.1. The Vascular Smooth Muscle of the Corpus Spongiosum Surrounding the Urethral Plate in Hypospadias Exhibited a Phenotypic Switch from a Contractile to a Secretory Type

**Figure 1:**
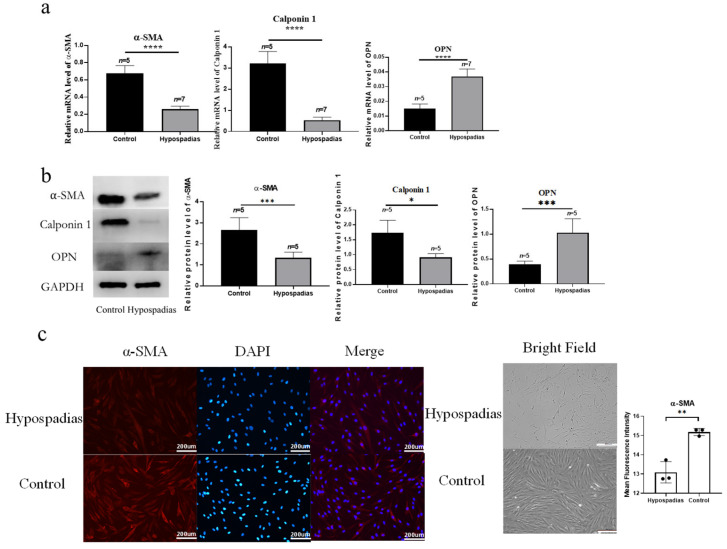
The vascular smooth muscle of the corpus spongiosum around the urethral plate exhibits a phenotypic switch from a contractile to a secretory type in hypospadias. Representative images are derived from corpus spongiosum tissues of hypospadias patients and controls included in the present study. (**a**) qRT-PCR and (**b**) Western blot of biopsies of corpus spongiosum around the urethra plate of hypospadias suggest that the transcription level and protein expression of α-SMA and Calponin 1 were lower than those of the control group, and the transcription level and protein expression of secretory OPN were higher than those of the control group. (**c**) In primary culture, VSMCs of corpus spongiosum around the urethral plate in hypospadias (*n* = 3) and control groups (*n* = 3) were cultured. After 20 days of primary culture, cells were observed as long spindle-shaped under bright-field microscopy, and α-SMA immunofluorescence staining was positive in both groups, consistent with the characteristics of smooth muscle cells. Quantitative analysis of α-SMA expression is shown on the right, including the mean fluorescence intensity (MFI), which was significantly lower in the hypospadias group than in the control group. The scale bar represents 200 µm in the image. The asterisks indicate statistical significance (* *p* < 0.05, ** *p* < 0.01, *** *p* < 0.001, and **** *p* < 0.0001).

qRT-PCR analysis revealed that the transcription levels of α-SMA and Calponin 1 were significantly reduced in the hypospadias group (*p* < 0.0001 for both), while OPN expression was markedly increased (*p* < 0.0001) (Figure 1a). Consistent with these results, Western blot analysis demonstrated decreased protein expression of α-SMA (*p* = 0.0007) and Calponin 1 (*p* = 0.0103), together with an increased expression of OPN (*p* = 0.0005) in the hypospadias group (Figure 1b). These findings suggest that vascular smooth muscle cells (VSMCs) in the corpus spongiosum surrounding the urethral plate of hypospadias undergo a phenotypic transition from a contractile to a secretory phenotype.

Primary VSMCs were successfully isolated and cultured from the corpus spongiosum tissues of both hypospadias and control groups. The cultured cells exhibited a spindle-shaped morphology, and immunofluorescence staining for α-SMA was positive in both groups, consistent with a smooth muscle-enriched population (Figure 1c). Quantitative analysis of mean fluorescence intensity (MFI) revealed significantly lower α-SMA fluorescence intensity in hypospadias-derived VSMCs compared with control-derived VSMCs (*p* < 0.01) (Figure 1c).

### 3.2. Identification of Pivotal Gene Modules Through WGCNA

**Figure 2:**
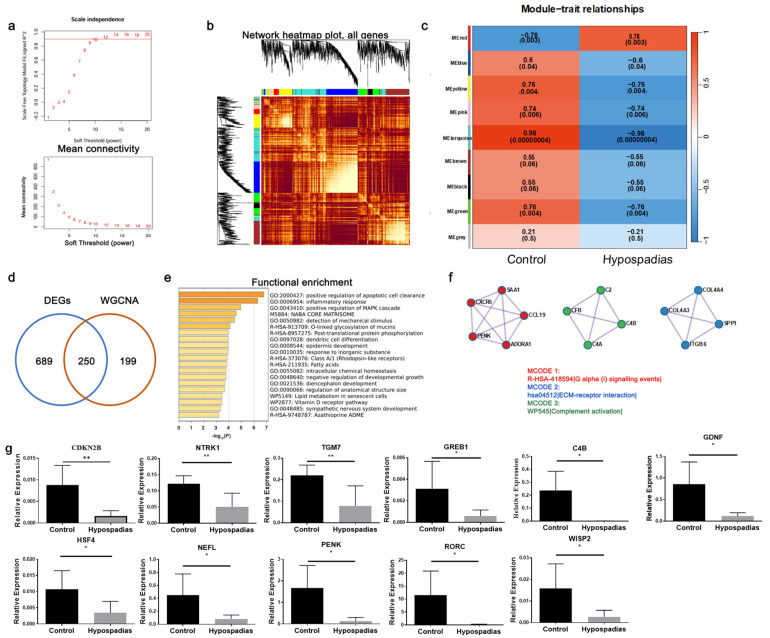
Weighted gene co-expression network analysis (WGCNA) was employed to identify gene co-expression networks in the corpus spongiosum of severe hypospadias. (**a**) The scale plot of WGCNA was utilized to determine the optimal vector power (cutoff value = 0.8). (**b**) the dendrogram generated through WGCNA depicted the clusters of genes that were differentially expressed, based on various metrics. Each branch of the dendrogram represented an individual gene, while the colors beneath the branches represented a co-expression module. (**c**) The heatmap depicted the association between gene modules and hypospadias phenotypes, with the correlation coefficient within each cube indicating the extent of correlation between gene modules and traits, exhibiting a gradient of decreasing intensity from red to blue. (**d**) A Venn diagram was employed to visualize the shared common hub genes between DEGs and WGCNA. (**e**) The turquoise modules’ hypospadias-related hub genes underwent functional enrichment analysis. (**f**) The present study identified the top Molecular Complex Detection algorithm (MCODE) terms associated with hub genes linked to hypospadias. A network was constructed based on protein-protein interactions (PPI) among hub genes related to hypospadias from the turquoise module. The MCODE algorithm was employed to identify the connected network components. (**g**) The RNA expression levels of GREB1, *C4B*, *GDNF*, *HSF4*, *NEFL*, *PENK*, *RORC*, *WISP2*, *NTRK1*, *TGM7* and *CDKN2B* were significantly different between control and hypospadias. The asterisks indicate statistical significance (* *p* < 0.05, ** *p* < 0.01).

To explore the gene co-expression network associated with VSMC phenotypic switching in hypospadias, Weighted Gene Co-expression Network Analysis (WGCNA) was performed using all samples with an optimal soft-thresholding power of 10 (Figure 2a). Hierarchical clustering generated multiple color-coded gene modules (Figure 2b), and module–trait correlation analysis identified a negative correlation between the turquoise module and the hypospadias phenotype (Figure 2c), indicating that genes in this module were downregulated in hypospadias tissues.

The turquoise module contained 449 genes, including *CDKN2B*, *GREB1*, *C4B*, *GDNF*, *NEFL*, *PENK*, *RORC*, *WISP2*, *NTRK1*, and *TGM7*, which were identified as hub genes. Overlap analysis between the DEGs and WGCNA-derived hub genes identified 250 common hub genes (Figure 2d).

Metascape analysis of these hub genes revealed enrichment in pathways associated with positive regulation of apoptotic cell clearance, epidermis development, and negative regulation of developmental growth (Figure 2e), indicating altered developmental signaling in the hypospadias corpus spongiosum. MCODE clustering further showed enrichment in Gα(i) signaling events, ECM–receptor interaction, and complement activation pathways (Figure 2f). Collectively, these findings suggest that the downregulation of hub genes in the corpus spongiosum of hypospadias patients may profoundly influence cellular developmental processes, particularly epidermal and extracellular matrix organization.

The qRT-PCR detection of these hub genes showed that the expressions of *GREB1*, *C4B*, *GDNF*, *HSF4*, *NEFL*, *PENK*, *RORC*, *WISP2*, *NTRK1*, *TGM7*, and *CDKN2B* were significantly decreased in the hypospadias group (*p* < 0.05) (Figure 2g). Further analysis of the literature revealed that *CDKN2B* regulates smooth muscle cell functions, such as accelerating VSMC proliferation, thereby affecting disease progression [8]. We performed further research on *CDKN2B* in VSMC phenotypic transformation behavior.

### 3.3. CDKN2B Expression Was Reduced in the Corpus Spongiosum Surrounding the Urethral Plate of Hypospadias

**Figure 3:**
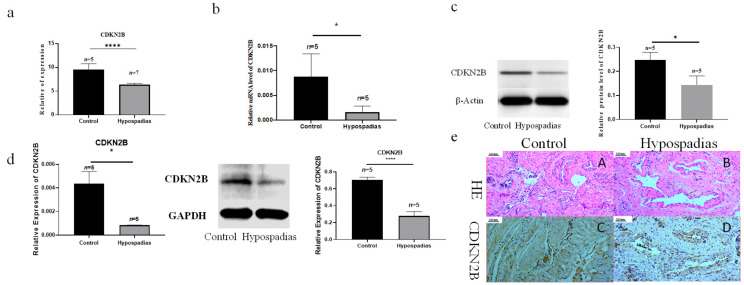
The expression of *CDKN2B* decreased in the corpus spongiosum around the urethral plate of hypospadias. (**a**) RNA-seq-based analysis of *CDKN2B* mRNA expression in corpus spongiosum tissues from control and hypospadias patients. (**b**) qRT-PCR validation of *CDKN2B* mRNA levels in corpus spongiosum tissues. (**c**) Western blot analysis of *CDKN2B* protein levels in corpus spongiosum tissues. (**d**) qRT-PCR and Western blot showing reduced *CDKN2B* mRNA and protein expression in corpus spongiosum VSMCs derived from hypospadias compared with control. (**e**) HE staining (**a**,**b**) and *CDKN2B* immunohistochemical staining (**c**,**d**) showing structural abnormalities and localization of *CDKN2B* in vascular smooth muscle layers. The scale bar represents 200 µm in the image. The asterisks indicate statistical significance (* *p* < 0.05, **** *p* < 0.0001).

RNA-seq analysis and subsequent validation revealed a significant reduction in *CDKN2B* mRNA expression in the corpus spongiosum tissues of hypospadias patients compared with controls (Figure 3a,b). qRT-PCR confirmed reduced CDKN2B transcript levels (*p* = 0.0084), and Western blotting demonstrated a corresponding decline in *CDKN2B* protein (*p* = 0.0241) in hypospadias corpus spongiosum (Figure 3b,c). Similar decreases in both *CDKN2B* mRNA and protein were observed in primary VSMCs isolated from the corpus spongiosum of hypospadias patients compared with control-derived VSMCs (Figure 3d).

Immunohistochemical staining and hematoxylin–eosin (HE) staining further revealed that *CDKN2B* was localized to vascular smooth muscle cells within the corpus spongiosum (Figure 3e). These findings indicate that *CDKN2B* expression is downregulated in the vascular smooth muscle of the corpus spongiosum surrounding the urethral plate in hypospadias.

### 3.4. CDKN2B Regulates Phenotypic Transformation of VSMCs in the Corpus Spongiosum

**Figure 4:**
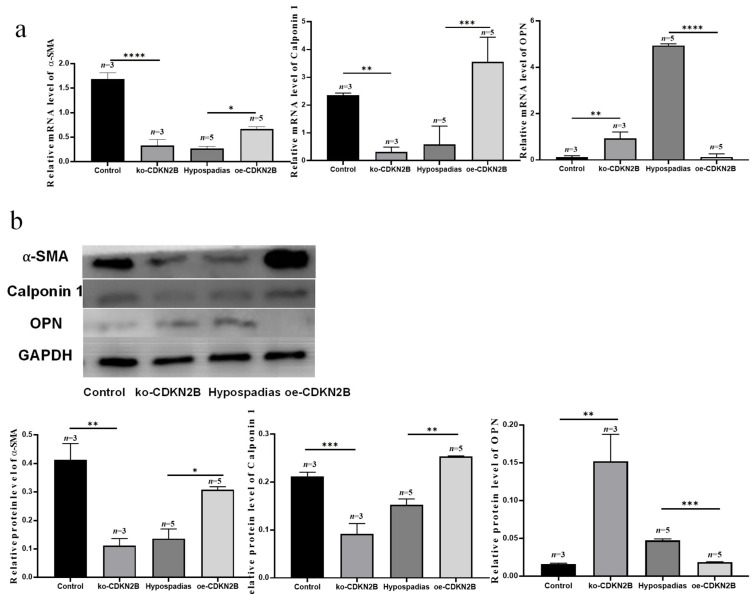
*CDKN2B* was involved in the regulation of VSMC phenotype transformation in corpus spongiosum. (**a**) qRT-PCR analysis of *α*-SMA, Calponin 1, and OPN expression after *CDKN2B* knockdown in control VSMCs and overexpression in hypospadias-derived VSMCs. (**b**) Western blot analysis of corresponding protein levels. The experimental groups were as follows: Control group (blank lentiviral control); ko-*CDKN2B* group (*CDKN2B* shRNA-infected controls); Hypospadias group (blank lentiviral infection in hypospadias-derived VSMCs); oe-*CDKN2B* group (*CDKN2B* overexpression in hypospadias-derived VSMCs). Values are the mean ± standard deviation. The asterisks indicate statistical significance (* *p* < 0.05, ** *p* < 0.01, *** *p* < 0.001, and **** *p* < 0.0001).

Knockdown of *CDKN2B* in control corpus spongiosum VSMCs (ko-CDKN2B group) resulted in decreased expression of contractile markers and increased expression of secretory markers (Figure 4). Specifically, qRT-PCR analysis showed that α-SMA and Calponin 1 transcripts were significantly downregulated (*p* < 0.0001 and *p* = 0.0060, respectively), while OPN expression was upregulated (*p* = 0.0060). Western blotting revealed corresponding decreases in α-SMA (*p* = 0.0014) and Calponin 1 (*p* = 0.0009) protein levels, alongside increased OPN expression (*p* = 0.0014).

Conversely, overexpression of *CDKN2B* (oe-CDKN2B group) induced the opposite effect: transcription and protein levels of α-SMA and Calponin 1 were significantly elevated (*p* = 0.0327 and *p* = 0.0004; *p* = 0.0117 and *p* = 0.0014), while OPN levels decreased (*p* < 0.0001 and *p* = 0.0004). These complementary loss- and gain-of-function experiments indicate that CDKN2B promotes maintenance of the contractile VSMC phenotype and suppresses the secretory phenotype in corpus spongiosum VSMCs.

### 3.5. CDKN2B Regulates the TGF/Smad and SRF/MYOCD Signaling Pathways to Inhibit VSMC Phenotypic Switching

Bioinformatic predictions and prior studies suggest that *CDKN2B* may regulate VSMC phenotype through *TGF/Smad* and *SRF/MYOCD* signaling pathways, as well as via transcription factors *FOXO4* and *KLF4* [9]. *TGF-β1* signals through *Smad2/3* to modulate ECM synthesis and VSMC differentiation, while SRF and its co-activator myocardin (MYOCD) drive transcription of contractile genes such as α-SMA and Calponin 1. We therefore examined these pathways in our model.

**Figure 5:**
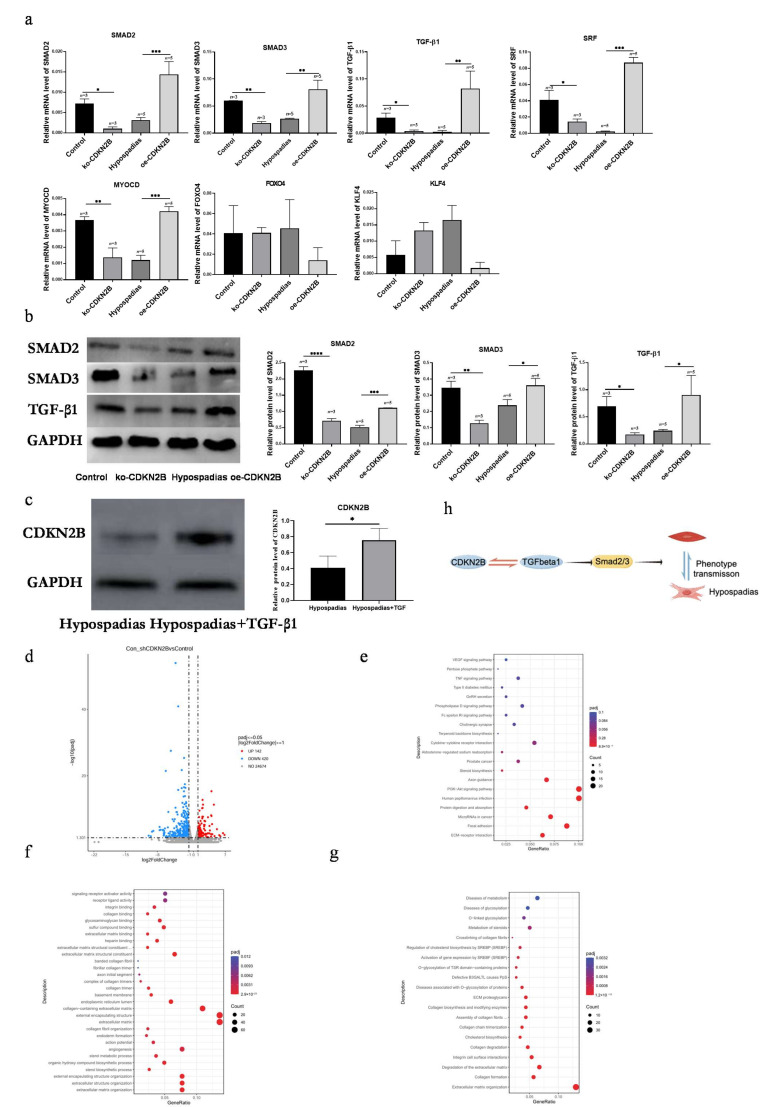
*CDKN2B* regulates TGF/Smad and SRF/MYOCD signaling pathways and inhibits the transformation of VSMCs from contractile to secretory phenotype. (**a**) qRT-PCR quantitative analysis of phenotype transformation related regulatory genes of urethral corpus spongiosum smooth muscle cells screened after lentivirus transfection; wherein *TGF-β1, Smad2, Smad3, SRF* and *MYOCD* were significantly different. But there was no significant difference between Foxo4 and KLF4 genes. (**b**) Western blot results of TGF/Smad pathway related to the phenotypic switching of urethral corpus spongiosum smooth muscle cells after lentiviral transfection. The expression of TGF/Smad pathway was down regulated after *CDKN2B* ablation, and increased after *CDKN2B* over-expressed. (**c**) Western blot results of *CDKN2B* related to the connection between *CDKN2B* and *TGF-β1*. The expression of *CDKN2B* was upregulated after *TGF-β1* induced. (hypospadias group vs. hypospadias + TGF-β1 group *p* = 0.0451). (**d**) the volcano map of differentially expressed genes of samples. Red dots are significantly upregulated genes, and blue dots are significantly downregulated genes. KEGG (**e**) and GO (**f**) and Reactome (**g**) enrichment analyses of the Control group of VSMCs and the ko-CDKN2B group. (**h**) The phenotype switch of urethral corpus spongiosum VSMCs was potentially mediated by the *CDKN2B-TGF/SMAD* signal pathway concluded in this study. Values are the mean ± standard deviation. The asterisks indicate statistical significance (* *p* < 0.05, ** *p* < 0.01, *** *p* < 0.001, and **** *p* < 0.0001).

qRT-PCR analysis revealed that the expression of *TGF-β1*, *Smad2*, *Smad3*, *SRF*, and *MYOCD* was significantly reduced in hypospadias VSMCs compared with controls (*p* < 0.0001), whereas *FOXO4* and *KLF4* expression showed no significant difference (*p* = 0.1995 and *p* = 0.0120, respectively) (Figure 5a).

Silencing *CDKN2B* led to decreased transcription of *TGF-β1*, *Smad2*, *Smad3*, *SRF*, and *MYOCD* (*p* = 0.0135, *p* = 0.0312, *p* = 0.0074, *p* = 0.0219, *p* = 0.0034), along with corresponding reductions in their protein levels (*p* = 0.0268, *p* < 0.0001, *p* = 0.0027) (Figure 5b).

In contrast, overexpression of *CDKN2B* significantly increased the transcription and protein expression of *TGF-β1*, *Smad2*, *Smad3*, *SRF*, and *MYOCD* (*p* ≤ 0.0010) (Figure 5b). Moreover, exogenous *TGF-β1* treatment upregulated *CDKN2B* expression in primary VSMCs derived from hypospadias tissues (Figure 5c), indicating reciprocal regulation between *CDKN2B* and the *TGF/Smad* signaling axis.

RNA-seq analysis of VSMCs between the control and ko-CDKN2B groups identified 562 DEGs, including 142 upregulated and 420 downregulated genes (adjusted *p* < 0.05) (Figure 5d). KEGG pathway analysis highlighted enrichment in VEGF signaling, cytokine–cytokine receptor interactions, and ECM–receptor interactions (Figure 5e). GO enrichment revealed involvement in extracellular matrix organization and encapsulating structure formation (Figure 5f), while Reactome analysis showed significant enrichment in collagen formation, collagen degradation, and integrin–cell surface interactions (Figure 5g).

Collectively, these findings indicate that *CDKN2B* inhibits VSMC phenotypic switching by modulating *TGF/Smad* and *SRF/MYOCD* pathways, thereby maintaining the contractile phenotype and suppressing pathological extracellular matrix remodeling.

## 4. Discussion

In summary, our findings demonstrate that downregulation of *CDKN2B* in vascular smooth muscle tissue of the corpus spongiosum from patients with hypospadias is associated with a phenotypic switch of vascular smooth muscle cells (VSMCs) from a contractile to a secretory state. This phenotypic transition contributes to the abnormal vascular composition of the corpus spongiosum in hypospadias. Mechanistically, this process appears to be mediated through the *CDKN2B–TGF/Smad* signaling axis (Figure 5h). Taken together, our findings indicate that CDKN2B plays a crucial role in regulating the phenotypic switch of corpus spongiosum VSMCs in hypospadias. This study therefore builds on our previous phenotypic observations [6] by providing a systems-level view of the regulatory network (via RNA-seq and WGCNA) and highlighting CDKN2B-centered signaling as a key mechanistic axis.

Hypospadias is a congenital malformation of the ventral penis characterized by incomplete development of the urethral spongiosum. Morphologically, the distal corpus spongiosum often presents as bifurcated columnar structures extending bilaterally along the urethral plate and merging with the glans penis. Histological observations by Baskin [3] and Putte [4] on fetal specimens confirmed the irregular dilation of vascular spaces in the urethral and penile spongiosum, consistent with aberrant tissue morphogenesis. Our previous work further showed that vascular abnormalities—such as loosely arranged vascular sinusoids, enlarged vascular cavities, and thickened vascular smooth muscle layers—are key features of the corpus spongiosum in hypospadias [5]. These findings collectively suggest that abnormal VSMC behavior plays a central role in hypospadias-related vascular malformation. Consistent with this, our earlier studies revealed a phenotypic transformation of spongiosal VSMCs from the contractile to the synthetic type [6].

VSMCs exhibit two interconvertible phenotypes: the contractile phenotype, which maintains vascular tone and structural stability under physiological conditions, and the secretory phenotype, which is characterized by enhanced extracellular matrix (ECM) synthesis and active proliferation. Contractile VSMCs express markers such as α-SMA and Calponin 1, whereas secretory VSMCs predominantly express OPN [10].

Under normal physiological conditions, VSMCs are primarily contractile; however, they retain remarkable plasticity and can transition between phenotypes in response to environmental cues. For instance, PDGF-BB promotes differentiation toward the contractile type, maintaining mature vessel integrity, while vascular injury or inflammatory cytokines—mediated through *KLF4* and other transcription factors—induce conversion to the secretory phenotype [11]. Excessive or dysregulated phenotypic switching contributes to disease progression, such as in atherosclerosis, where over-proliferation of secretory VSMCs thickens the vessel wall and increases secretion of inflammatory mediators like IL-1β [12].

In erectile dysfunction (ED), Lv et al. [13] and Zhang et al. [14] demonstrated that hypoxia and diabetes induce phenotypic transformation of penile cavernosal VSMCs, and that restoration of contractile markers α-SMA and Calponin 1 correlates with improved erectile function. Our study is the first to demonstrate that such VSMC phenotypic transformation occurs in hypospadias, a congenital developmental anomaly. Reduced expression of contractile markers (α-SMA, Calponin 1) and elevated expression of secretory marker OPN indicate a switch toward the secretory phenotype, which likely contributes to the abnormal vascular architecture of the corpus spongiosum in hypospadias.

RNA-seq analysis identified 1568 DEGs in hypospadias tissues, including 334 upregulated and 1234 downregulated genes. Enrichment analyses indicated significant involvement of pathways regulating protein metabolism and muscle structural organization, both crucial for VSMC phenotypic modulation (Figure S1). To refine the identification of functional gene modules, we applied Weighted Gene Co-expression Network Analysis (WGCNA), which has greater sensitivity than DEG analysis in revealing gene–trait correlations [15]. Importantly, by combining RNA-seq with WGCNA we were able to move beyond single-gene comparisons and identify gene modules most tightly associated with the hypospadias phenotype. The turquoise module showed a strong negative correlation with hypospadias (Figure 2c), and overlap analysis between DEGs and WGCNA hub genes (Figure 2d) highlighted *CDKN2B* as a pivotal hub gene within this module. This systems-level approach strengthens the argument that *CDKN2B* is not only differentially expressed but is embedded in a co-regulated network relevant to corpus spongiosum remodeling.

The INK4b-ARF-INK4a locus on chromosome 9p21, which includes *CDKN2B*, is strongly associated with coronary artery disease and atherosclerosis. *CDKN2B*, encoding the cyclin-dependent kinase inhibitor 2B, has been implicated in regulating VSMC proliferation and differentiation. Kojima et al. [16] reported that *CDKN2B* loss reduces Calponin synthesis, expands the lipid-rich necrotic core in plaques, and promotes the emergence of secretory VSMCs, thereby accelerating atherosclerosis. Similarly, our RNA-seq results revealed significantly lower *CDKN2B* expression in hypospadias, implicating its dysregulation in the abnormal development of the spongiosum vasculature.

In cardiovascular pathology, *CDKN2B* influences VSMC phenotype via *SRF* (serum response factor) and *MYOCD* (myocardin). Scruggs et al. [17]. demonstrated that reduced *CDKN2B* expression elevates *MRTFA*, a co-activator of *SRF/MYOCD*, leading to aberrant VSMC differentiation. *SRF* activates transcription of contractile genes through CArG-box binding [18]. while *MYOCD* acts as a co-activator that promotes expression of contractile proteins such as α-SMA, SM-MHC, SM22α, and Calponin 1. Reduced SRF levels, as observed in atherosclerotic plaques, impair cell cycle regulation and exacerbate vascular pathology [19]. Overexpression of *MYOCD* in cavernosal tissue of ED rat models restores contractile VSMCs and improves erectile function [14].

Consistent with these studies, we found that decreased *CDKN2B* expression in hypospadias correlates with reduced *SRF/MYOCD* signaling, diminished α-SMA and Calponin 1, and increased OPN, suggesting that loss of *CDKN2B* may drive VSMCs toward a secretory phenotype, contributing to vascular malformation of the corpus spongiosum.

Our findings further establish that *CDKN2B* modulates VSMC phenotypic switching through the *TGF-β/Smad* pathway. Li et al. [20] demonstrated high TGF-β1 expression in the urethral folds of male fetal rats, emphasizing its role in urethral fusion, while Zhou et al. [21]. showed that *TGF-β/Smad* signaling participates in epithelial–mesenchymal transition during urethral morphogenesis. *TGF-β* signaling is also essential for embryonic vascular development; its deficiency results in disorganized vasculature and abnormal vessel lumen formation [22]. In our study, overexpression of *CDKN2B* activated *TGF-β/Smad* signaling and upregulated contractile gene expression, promoting the secretory-to-contractile transition in VSMCs. These results reveal a previously unrecognized interaction between *CDKN2B* and *TGF-β/Smad* signaling in urethral vascular development, providing new insight into the molecular mechanisms underlying hypospadias-related vascular malformation.

Our study has several limitations. First, age-matched normal pediatric corpus spongiosum tissue is difficult to obtain, and our control samples were derived from older patients with traumatic urethral stricture. Although we sampled grossly normal tissue adjacent to the stricture and used the same processing procedures for both groups, age and clinical background differences could still influence gene expression profiles. Second, we defined primary cultures as VSMCs based on spindle-shaped morphology and strong α-SMA expression; however, α-SMA can also be expressed by activated fibroblasts, and a minor fibroblast contribution cannot be excluded. Third, our lentiviral experiments focused on *CDKN2B* knockdown in control-derived VSMCs and overexpression in hypospadias-derived VSMCs. Additional experimental groups—such as *CDKN2B* overexpression in control VSMCs and knockdown in hypospadias VSMCs—would provide an even more comprehensive assessment of *CDKN2B* function but were not feasible due to limited patient-derived material. Finally, in vivo functional validation in animal models is needed to confirm causality and to dissect temporal dynamics of CDKN2B-dependent signaling during urethral development.

Despite these limitations, our data identify *CDKN2B* as a novel molecular link between VSMC phenotypic switching and abnormal vascular remodeling of the corpus spongiosum in hypospadias and highlight the *CDKN2B–TGF/Smad–SRF/MYOCD* axis as a potential therapeutic target

## 5. Conclusions

In conclusion, our study reveals that *CDKN2B* plays a key regulatory role in maintaining the contractile phenotype of vascular smooth muscle cells in the corpus spongiosum. Downregulation of *CDKN2B* in hypospadias promotes VSMC phenotypic transformation through disruption of the *TGF-β/Smad* and *SRF/MYOCD* signaling pathways, leading to abnormal vascular remodeling and developmental defects of the corpus spongiosum. These findings identify *CDKN2B* as a novel molecular target involved in the pathogenesis of hypospadias and provide new mechanistic insight into vascular contributions to urethral malformation.

## Data Availability

All data generated or analyzed during this study are included in this published article. The raw data supporting the conclusions of this article will be made available by the authors on request.

## Supplementary Materials

The following supporting information can be downloaded at: https://www.mdpi.com/article/10.3390/biomedicines14010032/s1, Figure S1: RNA-seq of differentially expressed genes of corpus spongiosum tissue in hypospadias; Table S1: The qRT-PCR primer sequences were used in these research.

## Abbreviations

The following abbreviations are used in this manuscript:

VSMCs	Vascular smooth muscle cells
WGCNA	Weighted Gene Co-expression Network Analysis
ED	Erectile dysfunction
