# Supplementary material for: CDKN2B Inhibits Vascular Smooth Muscle Phenotypic Switching in Corpus Spongiosum Surrounding the Urethral Plate in Hypospadias
Source: Biomedicines. 2025 Dec 23;14(1):32. doi: 10.3390/biomedicines14010032 (PMC12839318; doi:10.3390/biomedicines14010032)
Supplement: Supplementary file 1 [file biomedicines-14-00032-s001.zip › biomedicines-3979730-supplementary.pdf]

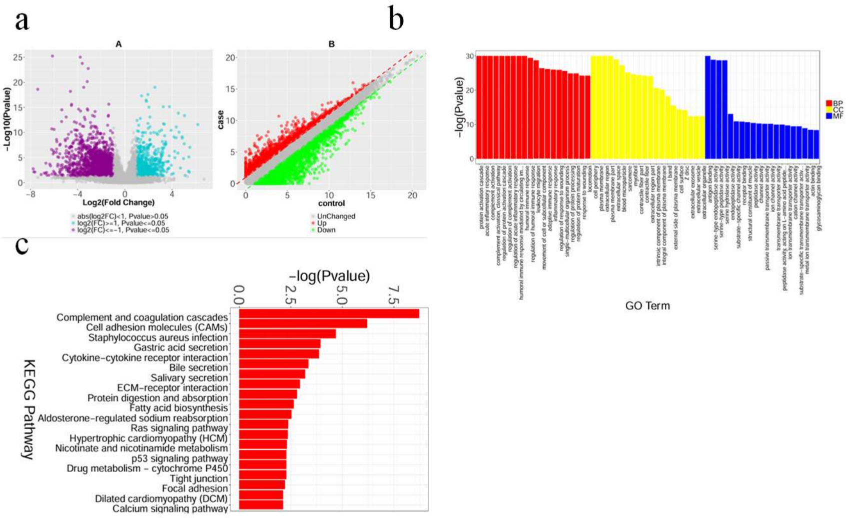

Figure S1. RNA-seq of differentially expressed genes of corpus spongiosum tissue in hypospadias

(a) the map of differentially expressed genes of samples: A is the volcano map, B is a scatter plot, (b) GO enrichment analysis of different genes in the corpus cavernosum of severe hypospadias. (c) KEGG pathway enrichment analysis of different genes in the corpus spongiosum of severe hypospadias.

Table S1: The qRT-PCR primer sequences were used in these research:

α-SMA	F, ATGCCAGTACACTGAATGATGG,<br>R, GATGCAGCATATACAGGAGCAA
TGF-β1 (Homo sapiens transforming growth factor, beta 1)	F, CAATTCCTGGCGATACCTCAG<br>R, GCACAACTCCGGTGACATCAA
FOXO4 (Homo sapiens forkhead box O4)	F, CCGGCAAAAGCTCTTGGTG<br>R, GGTCCACATATCGGCTTCTTCA
Calponin 1	F, CTGTCAGCCGAGGTTAAGAAC<br>R, GAGGCCGTCCATGAAGTTGTT
KLF4 (Homo sapiens Kruppel-like factor 4)	F, CGGACATCAACGACGTGAG<br>R, GACGCCTTCAGCACGAACT
OPN (Osteopontin)	F, TGAAATTCATGGCTATGGAA,<br>R, TGAAACGAGTCAGCTGGATG
SRF (serum response factor)	F, CCGGCAAGGCACTGATTCA<br>R, CTCATTCTCTGGTCTGTTGTG
MYOCD (myocardin)	F, CCACCTATGGACTCAGCCTAC<br>R, CTCAGTGGCGTTGAAGAAGAG
SMAD2(Homo sapiens SMAD family member 2)	F, TCATAGCTTGGATTTACAGCCAG<br>R, TTCTACCGTGGCATTTCGGTT
SMAD3 (Homo sapiens SMAD family member 3)	F, GCGTGCGGCTCTACTACATC<br>R, GCACATTCGGGTCAACTGGTA
